# Gabapentin and Post Tonsillectomy Pain-The Next Best Thing?

**DOI:** 10.5812/atr.9938

**Published:** 2013-02-01

**Authors:** Albert Moore

**Affiliations:** 1 Department of Anaesthesia, McGill University, Montreal, Canada

**Keywords:** Gabapentin, Tonsillectomy, Pain

*Dear Editor,*

Tonsillectomy is the second most common childhood ambulatory surgical procedure, and in the United States alone it is performed in over half a million children a year (1). The two major indications for tonsillectomy are recurrent tonsillitis or tonsillar hypertrophy that causes airway obstruction and sleep-disordered breathing. Tonsillectomy is associated with multiple postoperative complications, some of which are potentially life threatening. Bleeding is a common complication post-tonsillectomy and can lead to hospitalization in up to 4% of cases (2). Postoperative pain is also common, can be severe, and can lead to poor oral intake, dehydration and possibly weight loss if prolonged. Post-operative nausea and vomiting can occur in up to 70% of patients and may contribute to dehydration, or disruption of the surgical site if severe retching or vomiting occurs (1). The potential tonsillectomy complications make optimal postoperative management of tonsillectomy difficult. Adequate pain control is required to ensure rapid return to oral intake, and hospital discharge, but the analgesics commonly utilized after tonsillectomy can actually increase the risk of postoperative complications. Non-steroidal anti-inflammatories increase the risk of postoperative bleeding (3). Opioid medications increase the risk of nausea and vomiting, and also the ominous risk of respiratory depression in the patients with sleep disordered breathing. Acetaminophen alone provides less pain relief than opioids, and requires more rescue analgesia (4). An effective analgesic that does not increase the risk of postoperative bleeding, nausea, vomiting, or respiratory depression would be extremely useful in the management of tonsillectomy patients. Gabapentin may be a medication that meets these requirements. An analogue of γ-aminobutyric acid, it was introduced in the early 1990’s as an anti-epileptic medication. Although it never enjoyed much success in the prevention or treatment of seizures, gabapentin was soon found to have analgesic properties that made it an effective modality in the treatment of chronic neuropathic pain. Further investigation demonstrated its effectiveness in acute postoperative pain (5). Gabapentin has been shown to decrease the acute postoperative pain scores and opioid requirements in multiple surgical procedures, including hysterectomy (6), thyroidectomy (7), cholecystectomy (8), caesarean delivery (9), and various orthopaedic procedures (10, 11). Gabapentin may also have a role in preventing the development of chronic postoperative pain (12), and for reducing perioperative anxiety (10). Gabapentin is thought to exert its effect through the interaction with the α2δ subunit of voltage-dependant neuronal calcium channels, and its side effects seem to be related to the central nervous system, which includes increased sedation and dizziness. Because its mechanism of action does not involve the coagulation system, it does not seem to have an effect on bleeding complications. It may reduce the rates of nausea and vomiting after surgery (13), possibly due to its opioid sparing effect. It has been shown to reduce rates of other opioid-related side effects including pruritis (14). In reviews of its perioperative usage, it has not been reported to cause respiratory depression (5). Although gabapentin does appear to be a useful adjunct for the treatment of acute postoperative pain, the optimum timing, dose and the surgical procedures that will most benefit are currently not known. Gabapentin has been used in doses of 300 - 1200 mg, and has been given only preoperatively, or throughout the perioperative period. Although gabapentin is thought to be more effective when given at least one hour before the surgical insult, and perhaps is more effective when continued postoperatively, this remains to be confirmed.The potential usefulness of gabapentin in post-tonsillectomy management has been recognized by several investigators. Mikklesen compared preoperative and postoperative gabapentin to placebo in 49 patients, all of whom received rofecoxib (15). They found that gabapentin was associated with a significant reduction in opioid requirements postoperatively. However, they also found increases in dizziness, gait disturbances, and vomiting, which may be due to the fact that patients in the gabapentin group received 1200 mg before surgery, and 1800 mg daily for five postoperative days. Jeon et al. compared preoperative only gabapentin, to placebo in 58 patients undergoing tonsillectomy (16). They found that dynamic pain was decreased by one point on a visual analogue scale in the first 2 postoperative hours and that opioid requirements were significantly reduced in the gabapentin group. They did not find any between group difference in resting pain scores, nor in side effects including nausea, vomiting, sedation or dizziness. The study in this journal by Mogadam furthers the study of gabapentin for postoperative tonsillectomy pain by comparing the effect of gabapentin to diclofenac or placebo (17). The authors demonstrated a modest decrease of 1 point in visual analogue pain scores in the gabapentin group as compared to the placebo group 2 hours post-surgery. Although this reduction in pain scores might be considered clinically insignificant, it was accompanied by a reduction of mean meperdine requirements by about 15 mg, and an almost 2 hour earlier commencement of oral intake. The diclofenac group had a similar change in pain scores, opioid requirements, and oral intake. There was no difference found in vomiting, or dizziness between groups, and sedation was not determined. Although the results of this study are promising, they must be interpreted with caution, as it does have limitations. Blinding to treatment allocation was not reported, which if not present could have led to bias. It is unclear if patients had to spontaneously complain of pain to receive postoperative meperdine. This is important, as gabapentin may increase postoperative sedation, and lead to decreased requests for analgesics, not because of decreased pain, but increased sedation. As sedation was not assessed in this study, it is unknown what role it played in requests for analgesics. It is unclear if standard criteria were used to assess readiness for oral intake, so the earlier oral intake in the gabapentin group must be interpreted with caution. Statistical weaknesses including the lack of corrections for multiple comparisons also make the results less robust. Even taking into consideration the limitations of this study, gabapentin still shows promise as an analgesic for post tonsillectomy pain. However, there remains significant work to be accomplished before it should be introduced into the routine management of the tonsillectomy patient. The optimal dosing and timing must be determined. In addition, its effect on both rest pain, and swallowing induced pain should be further elucidated. Also, studies that are powered to adequately address the side effect profile must be undertaken, to determine the actual rates of sedation, dizziness, nausea and vomiting. In addition, the effects of gabapentin on the subset of patients who are undergoing tonsillectomy for sleep apnea must also be determined, as the increase in sedation may be more pronounced in these patients, and may increase their risk of respiratory depression. Also, the interaction of gabapentin with other medications used routinely in tonsillectomy must be assessed, including acetaminophen and dexamethasone. There remains significant work to be done to understand the effectiveness of gabapentin, but its potential to improve pain control without increasing the risk of post-tonsillectomy complications makes it an exciting possibility for the postoperative management of these patients.
